# Adult Ileocolic Intussusception from the Appendix

**DOI:** 10.1155/2019/3272618

**Published:** 2019-12-09

**Authors:** Natalie Green, William Krantz, Allison Tadros

**Affiliations:** ^1^West Virginia University School of Medicine, 1 Medical Center Dr., 26506 Morgantown, WV, USA; ^2^Department of Radiology, West Virginia University School of Medicine, 1 Medical Center Dr., 26506 Morgantown, WV, USA; ^3^Department of Emergency Medicine, West Virginia University School of Medicine, 1 Medical Center Dr., 26506 Morgantown, WV, USA

## Abstract

Intussusception is more commonly considered in the pediatric patient with abdominal pain, but can occur in adults as well. Adult patients are more likely to have an underlying intra-abdominal pathology leading to the condition. We present an adult patient presenting with abdominal pain with ileocecal intussusception diagnosed on imaging and confirmed surgically. In this case, appendiceal pathology served as the lead point for the intussusception.

## 1. Introduction

Intussusception occurs when one segment of bowel, the intussusceptum, telescopes or invaginates into an adjacent segment of bowel, the intussuscipiens, which can lead to obstruction or ischemia [1]. While intussusception is fairly common in children, affecting nearly 2000 children under 1 year of age each year in the United States, it is considerably rarer in adults, with only about 1% of all adult bowel obstructions being attributed to intussusception each year [2]. The clinical presentation of intussusception can vary significantly between children and adults, with children typically exhibiting classic symptoms including abdominal pain or cramping, bloody diarrhea, nausea or vomiting, and a palpable tender abdominal mass [3, 4]. Adults on the other hand will often exhibit chronic nonspecific symptoms including nausea, changes in bowel habits, and gastrointestinal bleeding [3]. The majority of adult intussusception patients also report pain as a major symptom, present in upwards of 90% of cases, with pain intermittency or periodicity being of significance due to the potential delay in diagnosis [5]. While only accounting for a small portion of bowel obstructions, the majority of adult intussusceptions are linked to underlying bowel pathologies [6], making it exceedingly important to promptly diagnose, and treat the intussusception, and its underlying causes. The present case describes an incidence of ileocolic intussusception secondary to acute appendicitis treated surgically with favorable outcomes.

## 2. Case Presentation

A 42-year-old female presented to the Emergency Department with a complaint of left lower quadrant abdominal pain and cramping that she reported began 2 days prior. Pain onset was followed by bloody stools, both of which resolved the following day aside with only some residual nausea. Abdominal cramping recurred early on in the morning of admission. The patient denied any ingestion of potentially contaminated water or unusual food. The patient history was also negative for recent surgeries, travel, or sick contacts. The patient reported no family or personal history of inflammatory bowel disease and had no other concerns aside from the left lower abdominal pain and bloody stools.

Upon examination, her vital signs revealed an elevated heart rate of 118 and a slight elevation in blood pressure at 134/88. Her abdominal exam showed normal bowel sounds and a soft abdomen but with mild diffuse tenderness. No rebound or guarding was noted.

**Figure 1:**
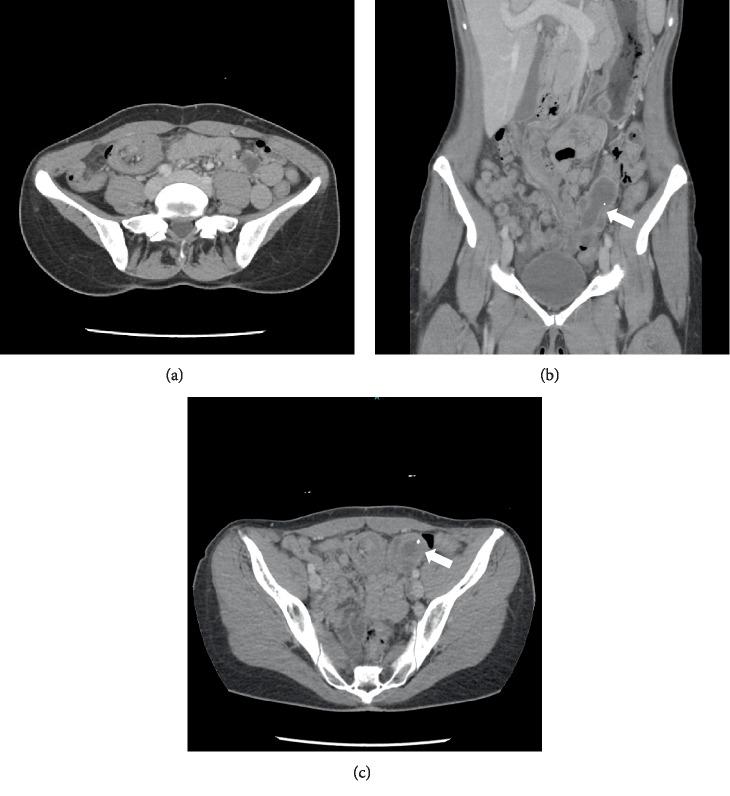
Axial and coronal CT images showing intussusception with the appendix indicated by the presence of a distended fluid-filled tube and an appendicolith (arrow).

Laboratory testing was largely unremarkable. CT abdomen pelvis with intravenous contrast revealed findings consistent with an ileocolic intussusception (Figure 1). A tubular structure was seen within the lumen of the intussusception which was determined to be a distended appendix potentially serving as the lead point. No indication of obstruction or bowel perforation was seen, but there was a small amount of intraperitoneal fluid. All organs were unremarkable aside from a non-obstructing stone seen in the right kidney.

The patient was admitted to general surgery for further evaluation and management. It was discovered that right colon and distal ileum had intussuscepted into the transverse colon, but it was unable to be reduced laparoscopically and therefore the procedure was converted to open laparotomy. After converting to an open procedure and mobilizing the colon, it was noted that a very long segment of colon had intussuscepted with a firm mass and lead point that had travelled into the transverse colon. The bowel was manually manipulated back to its original placement, at which point the base of the appendix appeared to be thickened and fibrotic, likely serving as the lead point for the intussusception. She also had a Meckel's diverticulum that was resected and sent to pathology. The patient was discharged the following day. Pathology revealed low-grade appendiceal mucinous neoplasm. A follow-up CT scan 6 months later revealed no evidence of disease.

## 3. Discussion

Intussusception occurs when a proximal segment of bowel prolapses into a distal or adjacent segment, which can lead to an obstruction and ultimately bowel ischemia, requiring surgical resection. Intussusception first described in 1674 by Paul Barbette of Amsterdam who described a case of intestinal invagination and suggested surgical reduction of the displaced bowel, but it wasn't until almost 120 years later in 1793 that John Hunter successfully described an intussusception in a postmortem specimen in London [3, 7]. In 1871, Sir Jonathan Hutchinson was officially the first surgeon to manually reduce an ileocolic intussusception in a 2 year old female, and soon after in 1876, Danish pediatrician Harald Hirschsprung published his experiences with nonoperative intussusception management using hydrostatic pressure enemas [7], a technique that is still used in pediatric cases [2–4].

The adult patient population accounts for anywhere from 5% to 10% of all cases of intussusception, with less than 5% of all adult intestinal obstructions being ascribed to intussusception [2–4]. Intussusceptions can be classified based on location, with entero-enteric being confined to the small bowel, colocolic in the large bowel, ileocolic between the terminal ileum, and ascending colon, and ileocecal in which the ileocecal valve actually serves as the lead point for the invagination [3, 4]. In clinical practice, it may be difficult to definitively discern an ileocolic from an ileocecal intussusception without surgical evaluation. A study of 745 adult intussusceptions reported that more than half (52%) were located in the small intestine, while only 17% were ileocecal like the case presented here [4]. In addition to being classified by location in the bowel, an intussusception can also be classified by etiology or root cause – idiopathic, benign, or malignant [5], and occasionally iatrogenic in patients with indwelling medical devices such as intestinal tubing [3].

In the adult population, less than 20% of intussusceptions are primary or idiopathic, with the majority being secondary to a pathologic lesion or irritant in the bowel lumen that is thought to lead to alterations in normal peristaltic activity [3, 5]. These abnormalities in the bowel wall will typically serve as the lead point for the intussusception as peristalsis will essentially “catch” the abnormal bowel and sweep it toward the adjacent segment leading to invagination. Adult risk factors typically include endometriosis, intestinal malignancies, Meckel's diverticulum or even appendiceal pathology as seen in this case. Unfortunately in adult cases, malignancies are the most common cause, with upward of 30% of small bowel intussusceptions being attributed to malignant lesions [3, 4] and over 60% in the large bowel; typically adenocarcinomas are the most common malignant lead point in the colon, while metastasis is more common in the small intestine [4, 8]. Because malignancies are commonly associated with intussusception in adults, close follow up is important.

Very few cases to our knowledge have been reported describing adult ileocolic intussusception with the appendix serving as the lead point. Honjo et al. published a case series of intussusception in adults and reported one myxoma of the appendix, two cystomyxomas of the appendix leading to ileocecal intussusception [8] Similarly, Kang et al. reported a case of appendicitis in an elderly patient resulting in cococolic intussusception [9]. In the present case, the patient was thought to have appendicitis, but was found to have low-grade appendiceal mucinous neoplasm on pathology.
